# Phylogenomic analysis of a methicillin-resistant Staphylococcus aureus ST764 isolate from Thailand in global context reveals limited cross-border transmission

**DOI:** 10.1099/mgen.0.001774

**Published:** 2026-07-16

**Authors:** Thidarat Netikul, Bharkbhoom Jaemsai, Wuthiwat Ruangchai, Thanakron Noppanamas, Sumalee Kondo, Lalita Narachasima, Prasit Palittapongarnpim

**Affiliations:** 1 Faculty of Medicine, Siam University, Bangkok, Thailand; 2 Department of Microbiology, Pornchai Matangkasombut Center for Microbial Genomics, Faculty of Science, Mahidol University, Bangkok, Thailand; 3 Faculty of Medicine, Thammasat University, Pathum Thani, Thailand; 4 Postgraduate Study, Faculty of Medicine, Thammasat University, Pathum Thani, Thailand

**Keywords:** arginine catabolic mobile element (ACME), evolutionary history, methicillin-resistant *Staphylococcus aureus* (MRSA), ST764, whole-genome sequencing

## Abstract

Methicillin-resistant *Staphylococcus aureus* (MRSA) ST764, a variant of ST5, has emerged and spread in Japan and China. We investigated the genome of a Thai isolate (SATU136) and the global phylogeny of ST764 to understand its global transmission history. The complete genome of SATU136 consists of a 2.89 Mb chromosome with a type II SCC*mec* element and a *qacB*-carrying plasmid but lacks the arginine catabolic mobile element (ACME). Based on the currently available global dataset, phylogenetic and phylodynamic analyses suggest that ST764 emerged in Japan in the early 1980s and was subsequently inferred to have disseminated to China and Thailand during the 1990s and early 2000s, coinciding with a peak in its overall effective population size. After this period, transmission was inferred to have become more geographically structured, with distinct clades forming in each country. Although cross-country transmission was inferred to be limited overall, onward dissemination to non-Asian countries was also detected within the Thailand-associated lineage. In contrast, the ACME element remained confined to a single Japanese subclade, with currently no evidence of spread beyond Japan. The relatively structured geographic distribution of ST764 offers a window for early detection upon introduction to new countries, which may facilitate control of its spread.

## Data Summary

All supporting protocols and data have been provided within the article and supplementary data. The long-read sequence data have been deposited in the NCBI Sequence Read Archive (SRA) under the accession number SRR34918246. The complete genome sequences of the chromosome and plasmid have been deposited to the GenBank database under accession numbers CP190357.1 and CP190358.1, respectively.

**Impact Statement**

Methicillin-resistant *Staphylococcus aureus* (MRSA) ST764 is an emerging healthcare-associated clone that has circulated mainly in Japan and China, with recent detection in Thailand. By generating the complete genome of a Thai isolate and analysing the global phylogeny, we demonstrate that ST764 likely originated in Japan before spreading to other countries. The finding that the arginine catabolic mobile element is confined to a Japanese subclade suggests that genomic diversification occurred prior to international dissemination. The absence of this element, which may contribute to community spread, likely limits the clone’s movement to transmission within healthcare settings. Early recognition of the introduction of this clone, together with strong infection control practices, may therefore reduce opportunities for wider spread. These results emphasize the importance of genomic surveillance to contain hospital-associated MRSA lineages.

## Introduction

Methicillin-resistant *Staphylococcus aureus* (MRSA) is an important aetiology of nosocomial infections. The spread of hospital-acquired MRSA (HA-MRSA) has been described worldwide, yet the community-acquired MRSA (CA-MRSA) and livestock-associated MRSA have been reported increasingly [1]. There have been several sequence types (STs) that are frequently associated with HA-MRSA worldwide, including ST5, ST8, ST22, ST239, etc. [2] Recently, we have reported MRSA ST764 as an emerging cause of HA-MRSA in a tertiary hospital in central Thailand [3], which was supported by a subsequent report from a surveillance study in another hospital in eastern Thailand, showing that it was the most common sequence type based on Multi-Locus Sequence Typing (MLST) [4].

*S. aureus* ST764, a single-MLST-locus variant of ST5, is a relatively recent genotype of MRSA originally discovered in Japan [5] and has spread mostly in the country and China [6]. It has been considered hypervirulent and is characterized by the abilities to produce high *α*-toxin levels, form biofilm, adhere to the cell and haemolyse red blood cells [7]. Moreover, a higher 30-day mortality rate among patients with bloodstream infections compared to other MRSA strains in Japan was recently revealed [8].

MRSA are generally resistant to *β*-lactam antibiotics due to the acquisition of staphylococcal cassette chromosome *mec* (SCC*mec*), a mobile genetic element carrying *mecA* and related genes. SCC*mec* has been differentiated into at least 14 types (SCC*mec*I to SCC*mec*XIV) according to the International Working Group on the Staphylococcal Cassette Chromosome elements (IWG-SCC) [9]. ST764 mostly carries SCC*mec* type II [5,10], similar to its ancestor, ST5, and occasionally carries SCC*mec* IV [11].

In contrast to the ST5 prototype strain, N315, and most ST5 isolates, some ST764 isolates were reported to harbour arginine catabolic mobile element (ACME), which was first described in the highly virulent CA-MRSA ST8 USA300 strain, which may contribute to community spreading [10,12]. This raises the concern for the possibility of the hypervirulent strain spreading outside hospitals. ACME is a mobile genetic element originally consisting of the *arc* gene cluster and the *opp* gene cluster, which encode for arginine deiminase and oligopeptide permease, respectively, promoting bacterial growth, virulence and transmission [13]. The ACME-*arc* system may help survival in acidic environments by converting arginine into ammonia, which allows the bacteria to grow on human skin (pH ~5.0) and, therefore, enhances skin colonization and host-to-host transmission. Apart from the one in ACME, *S. aureus*, including ST5 and ST764 strains, normally contains a native *arc* operon, whose functions have not been clearly differentiated from the ACME *arc* gene cluster yet. It is organized differently in the order of *arcABDCR*, encoding arginine deiminase, ornithine carbamoyltransferase, arginine–ornithine antiporter, carbamate kinase and a transcriptional regulator, respectively. Additionally, *argR*, which encodes the arginine repressor, is positioned upstream to *arcA* [14]. In the USA300 strain, the native *arc* gene cluster is located at the nucleotide positions 2,777,389–2,783,745, whereas ACME is located at positions 57,915 to 88,900, upstream of the SCC*mec* element at *orfX* integration site. It has been suggested that the integration of ACME occurred at the SCC*mec* attachment site, facilitated by SCC*mec* chromosome recombinases, as the direct repeats (DRs) of ACME were found close to an insertion sequence (IS) element of SCC*mec* [13]. Although the ACME *arc* operon shares similarity with the native *arc* operon, it has a different gene organization, as *arcADBC* and *argR* [15], encoding proteins with 79%, 82%, 57%, 47% and 48% identity compared to the native *arc* genes, respectively.

Based on the presence of *arc* and *opp* operons, ACME has been classified into a few distinct types: ACME I carries both operons, ACME II carries only the *arc* operon and ACME III contains only the *opp* operon. More recently, ACME types IV to VI have been proposed based on the presence of the potassium transporter-encoding *kdp* operon [16]. In ST764, a truncated form of ACME II (ACME II′) has been frequently identified [6]. The ACME *arc* gene cluster in ST764 is 6,174 bp long and 99.9% identical to the one of MRSA USA300 strain, albeit much shorter. The presence of ACME was initially considered a characteristic of MRSA ST764 reported from Japan [17]. However, it is absent in many isolates in a recent report [8] and is absent in all 52 ST764 isolates from China [7]. The emergence of ACME and the evolution of this MRSA genotype, therefore, needed further elucidation.

Previously, we reported the whole-genome sequencing data of five *S. aureus* ST764 isolates from Thai patients with invasive diseases. None of the patients had any known travel history to or epidemiological links with Japan or China, and this finding marked the first report of ST764 outside East Asia [3]. In the present study, we performed long-read sequencing on one of the isolates to generate a complete reference genome. We then conducted comparative genomic and phylogenetic analyses to characterize key genetic elements, including SCC*mec* and ACME, and to reconstruct transmission history and evolutionary dynamics of global MRSA ST764.

## Methods

### Bacterial samples used in the study

The *S. aureus* ST764 sample used in this study, designated as SATU136, was recovered from a blood specimen of an 80-year-old patient with a nosocomial infection. As reported in the previous study, the isolate was resistant to cefpirome and clindamycin, but susceptible to trimethoprim–sulfamethoxazole, linezolid, mupirocin and teicoplanin. The isolate was previously whole-genome sequenced using the Illumina NextSeq 500 short-read sequencing platform [3].

### Whole-genome sequencing

In this study, the bacteria were whole-genome sequenced with the Nanopore long-read sequencing platform. Genomic DNA was extracted using the QIAamp DNA mini kit (Qiagen, Hilden, Germany). DNA quality and quantity were measured with Nanodrop DenoVix® and the Qubit 3.0 fluorometer. The sizes of linear DNA fragments were determined by 1.0% agarose gel electrophoresis. The DNA was ligated as instructed by the manufacturer. Sequencing was then performed for ~72 h on a MinION Mk1B device using a MinION flowcell (FLO-MIN106 R9.4.1) (Oxford Nanopore Technologies, Oxford, UK). The genomic DNA input was 400 ng for each flow cell. Basecalling was performed by using the *basecalling* function of Dorado v1.0 with the super accurate (SUP) basecalling model.

### Genome assembly and sequence data analysis

The genome was firstly assembled from long-read data using Flye v2.9 [18] and then polished with Illumina short-read data which were reported previously using Pilon v1.24 [19]. The resulting polished genome sequence was reoriented to set the *dnaA* gene as the first position. The complete genome was subsequently annotated using the NCBI Prokaryotic Genome Annotation Pipeline (PGAP) [20].

Plasmid incompatibility group was identified using PlasmidFinder 2.1 [21]. Antibiotic-resistant genes were identified by the Comprehensive Antibiotic Resistance Database (CARD) Resistance Gene Identifier (RGI) and AMRFinderPlus [22,23]. Pathogenicity islands, mobile genetic elements and phage regions were predicted by Island Viewer 4, Alien Hunter, Phigaro and PHASTER, and their predictions were manually validated [24,27]. Virulence factor-encoding genes were identified by Virulence Factor Database (VFDB) [28].

### Genome comparison

The complete genome of SATU136 was compared to the complete genomes of the other available complete genomes of MRSA ST764, i.e. KUH180062 (accession number: NZ_AP020320.1) [29], N10CSA27 (accession number: NZ_CP094443.1) [7] and MRSA ST5 N315 (accession number: BX571856.1) [30]. The genome assembly of MRSA ST764 strain NN54 (accession number:NZ_BAFI00000000.1) [10], which was sequenced by pyrosequencing, was also compared.

The genomic comparison of selected regions was generated and visualized using Easyfig. Comparison of the *SCCmec* region of KUH180062, SATU136, N10CSA27 and N315 was done for the segments between *dnaA* (F6Y17_RS11945) and *helicase* (F6Y17_RS12500). Comparison and visualization of the chromosomes SATU136, KUH180062, NN54, N10CSA27 and N315, shown in Fig. 2, were performed using Proksee [31] with platform blastn [32], using SATU136 as the reference genome.

### Phylogenetic analysis

To examine phylogenetic relationships of SATU136 and other MRSA ST764 isolates from different geographical locations, we compiled a dataset of publicly available genomes of *S. aureus*. The fastQ data were queried from the NCBI SRA database using the species name, ‘*Staphylococcus aureus*’ and ‘strategy wgs’ as search terms. Up to August 2025, 196,788 samples were identified. The dataset was further filtered based on sequencing platform (illumina or bgiseq), library selection (random or pcr or rt-pcr), library source (genomic) and library layout (paired). The ST of the samples was then determined using stringMLST [33], which yielded 421 samples of *S. aureus* ST764. The assembled genome data were also retrieved from the NCBI RefSeq Assembly database, under the NCBI Genome database, using the species name. The ST of the samples was identified by fastMLST, resulting in 90 samples, including SATU136. The deduplication was done by verification of the biosample numbers of the dataset. When multiple samples shared the same collection date, the metadata, if available, was examined to confirm that they originated from different patient IDs. This finally resulted in a total of 511 samples with unique biosample identifiers, belonging to 46 BioProjects and reported in 28 publications as shown in Table S1(A, B) (available in the online Supplementary Material). Most of these projects were cross-sectional or surveillance studies, while two studies specifically investigated the transmission of ST764 isolates [7,34].

The samples were searched for the *mecA* gene by ResFinder [35] and revealed that all samples contained *mecA* and were presumed to be MRSA.

All MRSA ST764 genome sequences were mapped to the SATU136 and KUH180062 [29] genome sequences to generate two whole-genome alignments using Snippy [36]. The genomic sequence of *S. aureus* ST5 strain ATCC_700699 (MRSA Mu50) (SRR7829189) was included as the outgroup. Eighteen samples with missingness greater than 25% were excluded from the dataset, resulting in 493 ST764 samples for phylogenetic analysis: 255 from Japan, 217 from China, 15 from Thailand, 1 each from Australia and Russia, 2 from the UK and 2 from the Netherlands. The whole-genome alignment was used as input for detecting and masking recombinant regions using Gubbins [37], with the options --tree-builder iqtree, --best-model, --ufboot 1000 and --outgroup SRR7829189.

To time-calibrate the tree, we used tip-dating, which uses sample collection dates as calibration points at the phylogeny tips and fits a molecular clock model to convert branch lengths into time, enabling estimation of the substitution rate and dates of internal nodes including the tMRCA. We first tested for the presence of temporal signal in the dataset. Approximately 20% of samples had collection dates reported as a range of years; for these, the midpoint of each range was used as the tip date. For three samples with unknown dates, the publication date was used as the tip date (proper tip dates for these samples were subsequently estimated with Least-Squares Dating version 2 (LSD2). A root-to-tip regression analysis was then performed using the lm function in R, which revealed a moderate temporal signal (*R*^2^=0.42, *P*<0.001) (Fig. 3a). To assess the robustness of this approach, we additionally performed 1,000 replicate regressions in which collection dates were randomly assigned within their reported ranges, yielding *R*^2^ values highly consistent with the midpoint-based estimate (Fig. S2). To further confirm that the observed temporal signal was genuine rather than an artefact of sample composition, a date-randomization test was performed with 1,000 replicates, in which sampling dates were randomly permuted among tips and the substitution rate was re-estimated; the rate inferred from the observed dates fell well outside the distribution of rates obtained from the randomized replicates (Fig. S3), indicating that the temporal signal in the dataset is genuine rather than an artefact of sample composition. Together, these results confirmed a robust temporal signal in the dataset, warranting tip-dating analysis.

A time-resolved phylogeny was then inferred from the rooted tree and sample collection dates using LSD2 [38]. The recombination-free phylogeny from Gubbins (*.final_bootstrapped_tree.tre), in which branch lengths are reported as raw SNP counts, was used as input together with the sampling dates. LSD2 natively accepts heterogeneous date inputs, including exact dates, date ranges and upper or lower bounds (e.g. “before a specified year or after a specified year”), and infers dates for tips with unknown collection times by leveraging the temporal signal from dated tips. For samples with collection dates reported as a range of years, the full range was provided directly. For three samples with only an upper bound on the collection date (taken from the ‘First_created_date’ field recorded in NCBI SRA), this date was specified as the latest possible year (upper bound) of isolation. LSD2 was run under the temporal constraints mode with a relaxed lognormal clock (-q 1), branch-length-proportional variance estimation (-v 2) and 1,000 simulations for confidence interval (CI) estimation (-f 1000). Because the input branch lengths are in raw SNP counts, the substitution rate reported by LSD2 was rescaled to genome-wide units by dividing by the non-recombinant alignment length [2,686,208 bp; obtained by subtracting unique recombinant regions identified by Gubbins, totalling 212,103 bp (7.3% of the whole genome), from the whole-genome alignment of 2,898,311 bp]. The resulting clonal substitution rate was 9.14×10⁻⁷ substitutions/site/year (95% CI: 8.03×10⁻⁷–1.61×10⁻⁶), and the most recent common ancestor of *S. aureus* ST764 was estimated at 1981 (95% CI: 1966–1994). To assess robustness to reference genome choice, the entire workflow (Snippy for genome alignment, Gubbins for recombination masking and LSD2 for tip-dating) was repeated using a Japanese strain as an alternative reference (KUH180062). The resulting tree topology (Fig. S4), clonal substitution rate (9.17×10⁻⁷ subs/site/year, 95% CI: 8.21×10⁻⁷–1.65×10⁻⁶) and tMRCA 1981.69 (95% CI: 1967.34–1995.41), were concordant with the primary analysis.

Phylogeographical history was inferred from the time-calibrated tree (generated using SATU136 as the reference) and samples’ countries of collection using PastML with the marginal posterior probability approximation (MPPA) method and the F81 substitution model [38]. Bacterial effective population size was estimated from the time-calibrated tree using the *mlskygrid* function of the mlesky [39]. The number of grid points (res) was set to 30, determined as the optimal value by the *optim_res_aic* function. The smoothing parameter (tau) was set to 9.14, given the res value.

### Detection of ACME

The presence of ACME in each genome was detected by two complementary approaches: (1) blast searches based on the *arcA* gene from the ACME of strain KUH180062 as a representative marker or (2) ACME data retrieved from previously published studies when available.

### Data availability

Long-read sequence data of SATU136 were deposited in the SRA repository of NCBI under the accession number SRR34918246. The complete genome sequence of SATU136 was deposited to the GenBank database under accession numbers CP190357.1 (chromosome) and CP190358.1 (plasmid), respectively.

## Results

### Complete genome of SATU136

A hybrid assembly of the SATU136 genome, using long-read data from this study and previously reported short-read data [3], yielded a circular chromosome of 2,898,311 bp with a G+C content of 32.9 mol%. Annotation using the NCBI PGAP [20] predicted the presence of 2,953 genes, comprising 2,784 coding sequences (CDSs), 79 RNA genes and 90 pseudogenes. In addition to the chromosome, the isolate harboured a 35,433-bp-long plasmid, designated as pSATU136, as shown in Fig. S1. The plasmid belongs to the lnc18 incompatibility group and contains 45 predicted CDS. A blast search revealed that pSATU136 shares high sequence similarity with plasmid pTZ2162 (accession no. AB304512.1), showing >99% nucleotide identity across 35 kb homologous regions. Plasmid pTZ2162 was previously reported in MRSA ST5 from Japan [40].

### SCC*mec* element and ACME

**Fig. 1.**
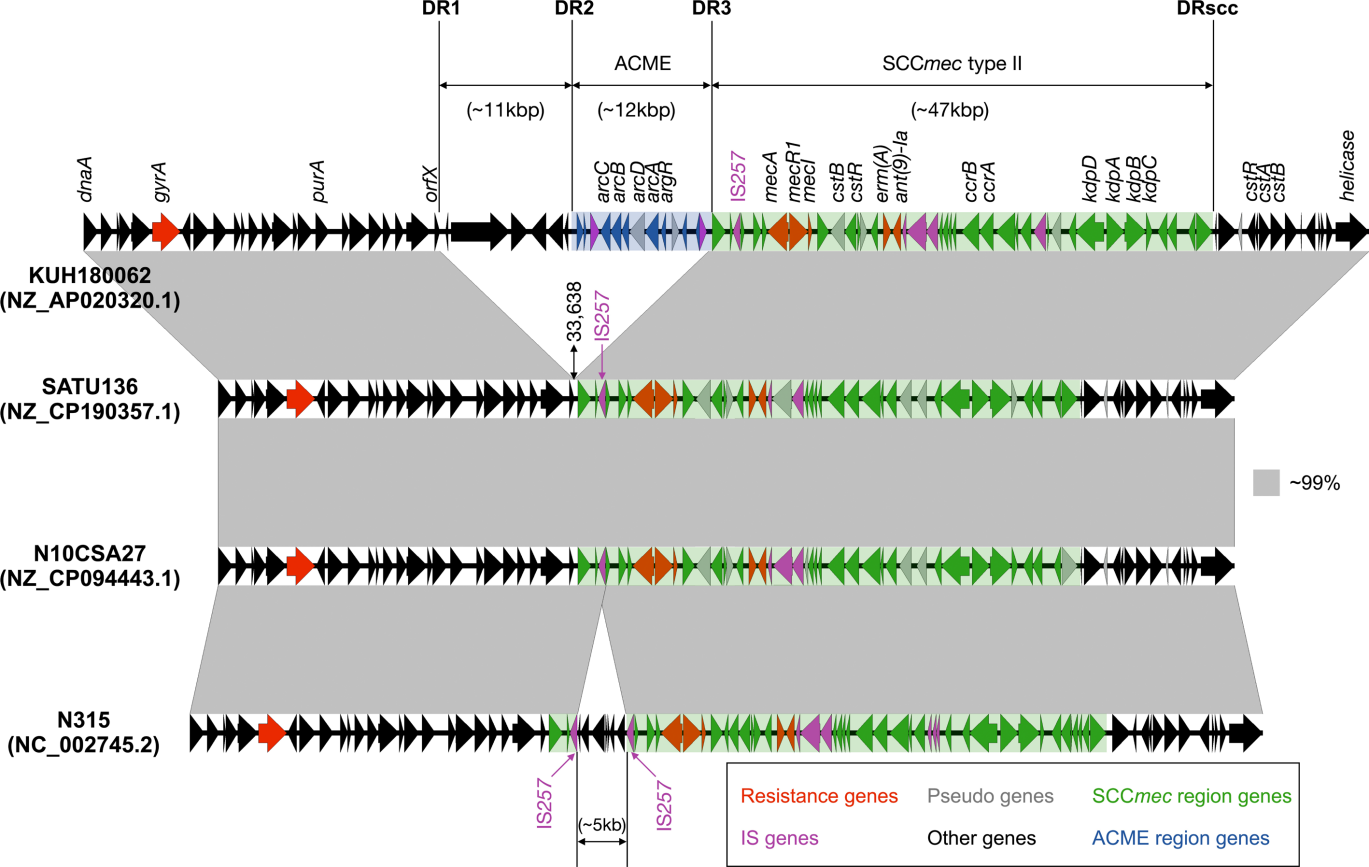
Comparative analysis of the SCC*mec* and ACME genomic regions. Linear comparison shows the gene organization of the MRSA ST764 SATU136 strain with the Japanese ACME-positive strain KUH180062, the Chinese ACME-negative strain N10CSA27 and the ST5 N315 strain. The different segments with the size less than 1,000 bp are not shown. Genes are represented by arrows indicating their orientation, coloured by functional category (see legend). A grey shading between the strains indicates nucleotide similarity. The upper vertical lines indicate the locations of DRs: DR1, GAAGCGTACCACAAATAA; DR2, GAAGCGTATCATAAGTGA; DR3, GAAGCATATCATAAATGA; and DR_SCC_, GAGGCGTATCATAAGTAA.

The SATU136 genome lacks the ACME element, containing only the native *arc* gene cluster, *arcAFDCR* (*arcF* is homologous to *arcB*), at position 2,804,268 to 2,809,828. Compared to the ACME-positive strain KUH180062 [29], SATU136 has a deletion of ~25 kb at the ACME region. Nevertheless, both strains exhibited high similarity in the regions flanking this deletion (Fig. 1).

The ACME-SCC*mec* region of KUH180062 contains four DRs. The first, DR1 (GAAGCGTACCACAAATAA), is situated within the distal end of the *rlmH* gene, hereafter referred to as *orfX*. The second and third repeats, DR2 (GAAGCGTATCATAAGTGA) and DR3 (GAAGCATATCATAAATGA), flank the ACME region. The final repeat, DR_SCC_ (GAGGCGTATCATAAGTAA), is located at the terminus of the SCC*mec* element.

In contrast, the SATU136 genome contains only DR3 and DR_SCC_, which flank its 47.6-kb-long type II SCC*mec* element. A comparative analysis with the *S. aureus* ST5 reference strain N315 revealed the absence of a 5.4 kb segment from the SCC*mec* region of the SATU136 (Fig. 1), which was also deleted from the KUH180062 and N10CSA27 genomes. The segment carried CDSs for a truncated *repB* plasmid replication protein, two distinct plasmid recombination enzymes, two hypothetical proteins and the drug resistance genes *bleO* and *addD*, which confer resistance to bleomycin and kanamycin, respectively. In N315, this region is flanked by two IS*257* elements, whereas only a single IS*257* element is present in SATU136. This suggests that the deletion occurred via homologous recombination between the two IS*257* elements.

### Antimicrobial resistance genes

Identification of antibiotic resistance genes in the SATU136 chromosome was conducted using AMRFinderPlus [23], which reported genes highly similar to the reference sequences, mostly exceeding 99%. The fluoroquinolone resistance was identified by CARD RGI [22]. Beyond the *mec* operon, which was present in every MRSA isolate, SATU136 harboured several antibiotic resistance genes commonly reported in MRSA ST764, including the ones for macrolides (*ermA*), aminoglycosides [*ant* (9)-Ia], tetracyclines [*tet(M*)] in Tn*5251*, fosfomycin (*fosB*) and fluoroquinolones (*gyrA* S84L, *parC* S80Y) in the chromosome, as shown in Table S2. Similar to MRSA ST5 N315, three copies of *ermA* and *ant* (9)-Ia genes were located in Tn*554* regions: Tn*554mec*, Tn*554a* and Tn*554b1*. Nevertheless, *mupA* that confers resistance to mupirocin was not found in SATU136, correlating with the susceptible phenotype [3]. pSATU136 carried the other common antibiotic resistance genes to ST764, including the ones for aminoglycosides (*aac(6′)-Ie/aph(2″)-Ia*), fosfomycin (*fosD*), quaternary ammonium compound (*qacB*) and beta-lactamase operon (*bla1*, *blaZ* and *blaR1*) similar to pTZ2162 [40]. Previous studies have shown that bacteria harbouring pTZ2162 exhibited increased resistance to fosfomycin and aminoglycosides. The QacB protein has been associated with decreased susceptibility to disinfectants and fluoroquinolones [40,41].

### Pathogenicity islands, ISs, prophages and virulence genes

**Fig. 2.**
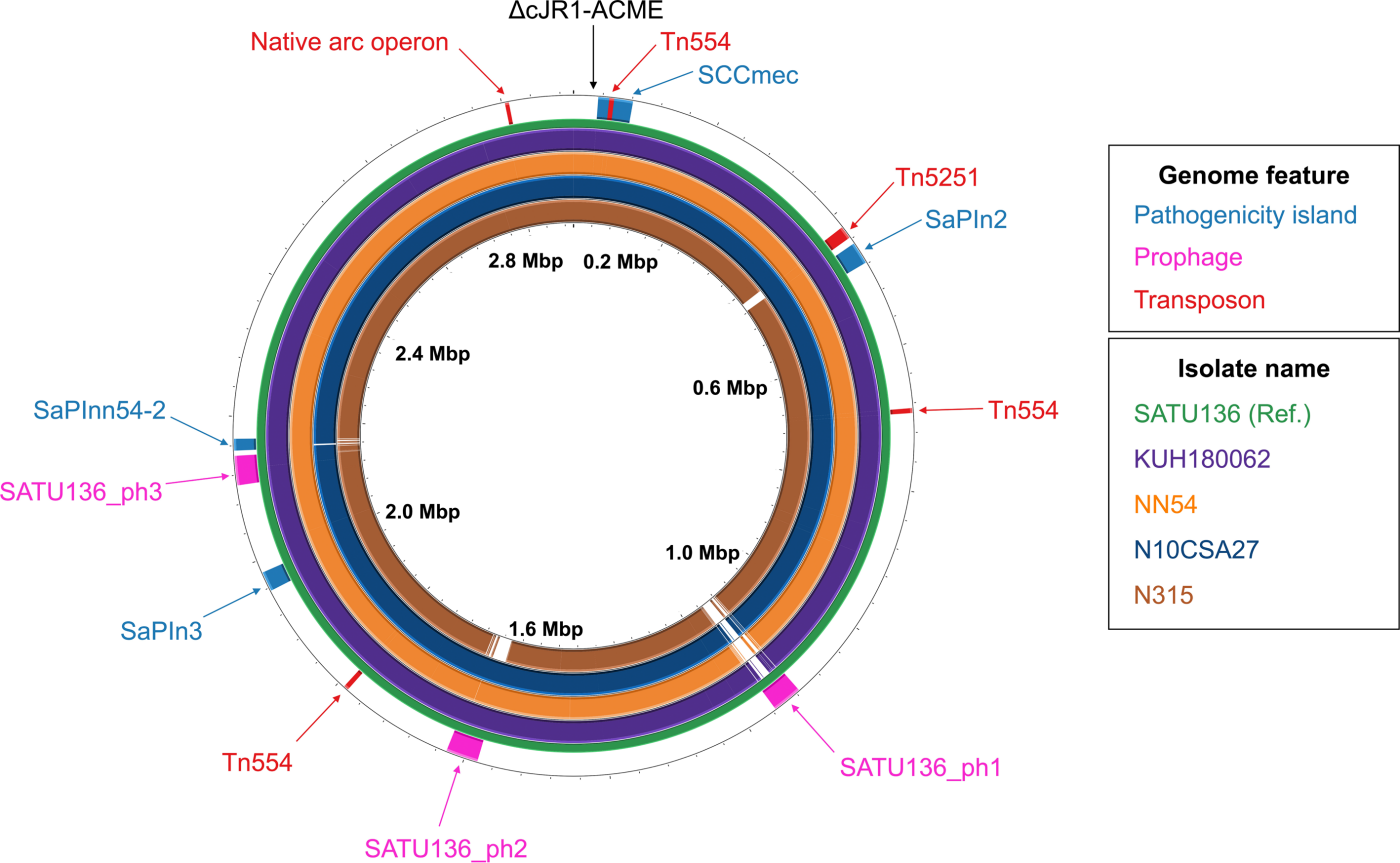
Chromosomal comparison. The genomes of ST5 N315, ST764 NN54, the Japanese ACME-positive strain KUH180062 and the Chinese ACME-negative strain N10CSA27 were compared using ST764 SATU136 as the reference genome, shown as the green circle. The white areas in the circles represent the absence of the DNA regions compared to the SATU136 genome. The outer circle indicates the positions of SCC*mec*, pathogenicity islands, prophages and transposons, highlighted in different colours. SATU136 and N10CSA27 lack ACME, the position of which in NN54 and KUH180062 is indicated by the black arrow. The prophage SATU136_ph1 was absent in other isolates.

SATU136 possessed the exotoxin island SaPIn2 and the enterotoxin island SaPIn3, members of νSa*α* and νSa*β*, respectively, but lacked the toxic shock syndrome toxin 1 island, SaPIn1 (Fig. 2). Similar to MRSA N315, SATU136 SaPIn2 comprised the clusters of exotoxin genes (*set6-set15*) and lipoprotein-like genes (*lpl1-lpl9*), while SaPIn3 comprised clusters of serine protease genes, leukotoxin genes (*lukD* and *lukE*) and enterotoxin genes (Table S3). The third pathogenicity island in SATU136, SaPInn54-2, is a 16.1-kb-long ST764-specific island that was first described in NN54, another ST764 strain [10], and flanked by 20 bp *att* sequences (TTTTACATCATTCCCGGCAT). SaPInn54-2 appeared to be a mosaic of two SaPIs, consisting of a 7-kb-long *fhuD* (iron-hydroxamate ABC transporter)*-*containing segment reported from an MRSA ST239 strain OC3 [42] and an 8-kb-long segment reported from a Japanese isolate, strain no. 10 [43]. However, unlike the latter, the corresponding region in SaPInn54-2 does not contain the staphylococcal enterotoxin B gene (*seb*) [43]. NN54 contained another *seb*-carrying island, called SaPInn54 [10], which was completely absent in SATU136.

Three prophage regions, each ~40 kb in length, were identified and designated as SATU136_ph1, SATU136_ph2 and SATU136_ph3 (Table S4). SATU136_ph1 was absent in N315 and KUH180062 (Fig. 2), while both latter contained SATU136_ph2 and SATU136_ph3. SATU136_ph1 was partially similar to the phage SP6, isolated from animal farm soil in Korea, and the well-characterized phage 53 [44,45]. SATU136_ph2 was most similar to the phage φSa2wa_st72 (accession no. MG029513) but lacked the Panton–Valentine leukocidin (PVL) gene [46]. SATU136_ph3 showed partial similarity to φN315 and carried the staphylokinase (*sak*) gene [30].

VFDB [28] identified all classes of virulence factors in SATU136, including adhesion-related proteins, enzymes, immune evasion-related proteins, secretion systems and toxins (Table S5). The majority of these virulence factors were similar to those found in N315, and neither strain carried the cytotoxin gene encoding PVL. Notably, the toxic shock syndrome toxin-encoding gene was also absent in SATU136.

### Global phylogeography and phylodynamics of MRSA ST764

A comprehensive search was performed to compile all available genome sequences of *S. aureus* ST764 from all geographical locations, resulting in a curated dataset of 493 ST764 samples (Table S1). We used SATU136 as the reference genome for whole-genome alignment. The resulting alignment was then used to infer a maximum-likelihood tree using Gubbins [37], which identifies and excludes recombinant regions before inferring the phylogeny from the recombination-free SNP alignment. Based on the basal branching pattern and ultrafast bootstrap (UFBoot) scores, the samples formed five major clades, each containing at least ten samples (clades I–IV), all with high UFBoot scores (≥98) except for clade II, which had a UFBoot score of 73 (Fig. S4A). Among these, clade I is the largest (*N*=259), containing samples from Japan and China that form four subclades, each composed exclusively of samples from either Japan or China. Clade IV (*N*=89) contains samples from multiple countries, with the majority from Japan (*N*=69). Nested within clade IV is a subclade (*N*=20) dominated by samples from Thailand (*N*=15), which itself contains a nested ‘Non-Asian subclade’ comprising samples from the UK (*N*=2), Russia (*N*=1), the Netherlands (*N*=1) and Australia (*N*=1) (Figs S4 and S5). Repeating read mapping with an additional reference, KUH180062 (Japan), and inferring an ML phylogeny with Gubbins yielded a largely consistent topology. However, the basal branching patterns differed and exhibited low UFBoot scores, indicating that the basal branches could not be reliably resolved (Fig. S4). Nevertheless, the composition of each subclade was highly consistent across the two phylogenies, with strong branch support.

To reconstruct the bacterial transmission history, an evolutionary timescale was first estimated using a tip-dating analysis using the results that had SATU136 as reference. The resulting time-resolved phylogeny was then used to infer transmission history using ancestral state reconstruction.

Tip-dating analysis of our curated dataset estimated the time to the most recent common ancestor (tMRCA) of MRSA ST764 to 1981 (95% CI: 1966–1994), earlier than previously reported by Hisatsune *et al*. [8].

**Fig. 3.**
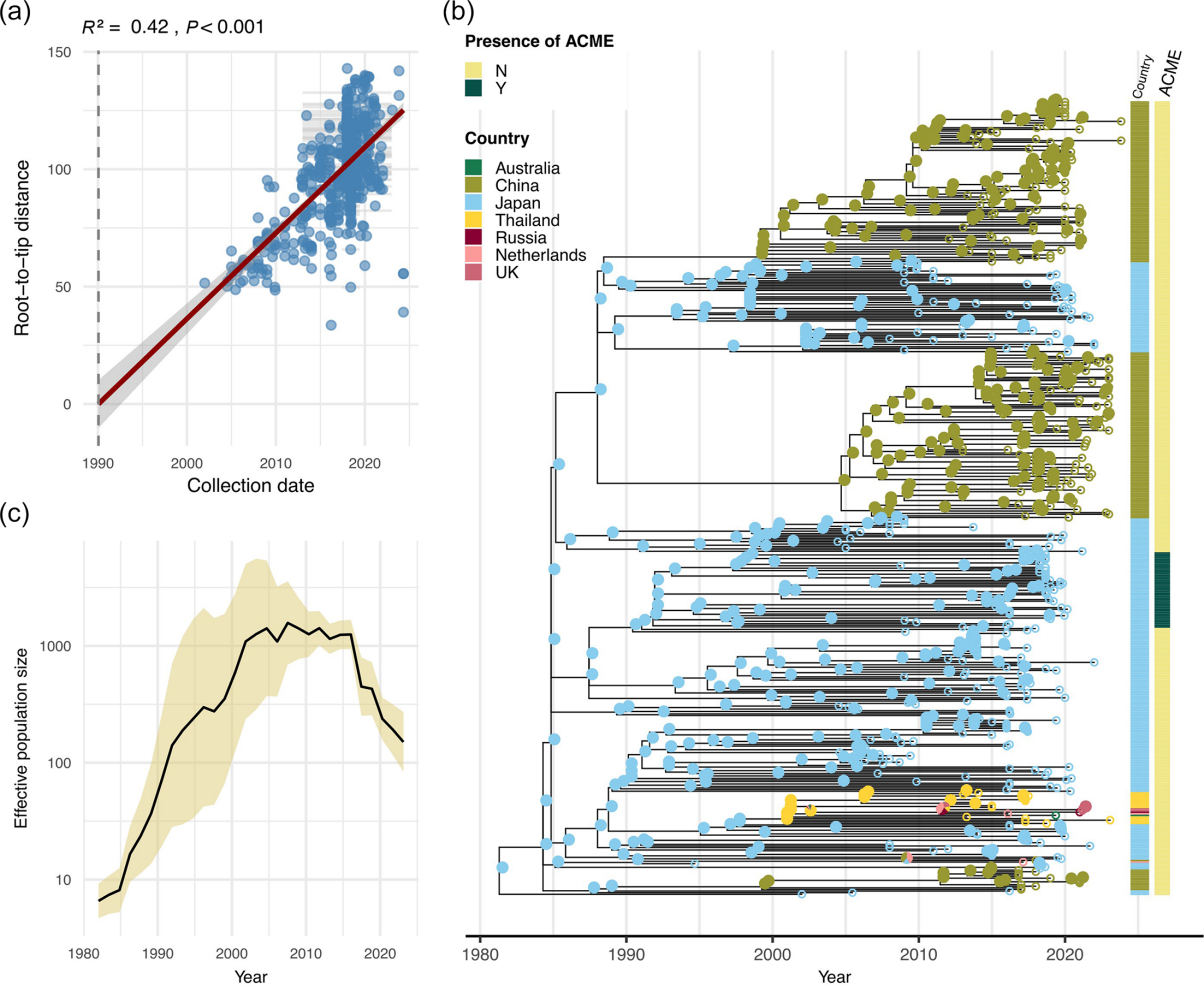
Time-resolved phylogeography and phylodynamics of global MRSA ST764. (a) The plot between root-to-tip distance and the year of collection. (b) Time-calibrated phylogeny of global MRSA ST764 with estimated tMRCAs and 95% CI of major clades are shown. Coloured bars, from left to right, indicate the presence of ACME and the country of collection (see legend). Coloured dots on each internal node show the marginal probability of being in different geographical locations estimated using the MPPA method with the F81 substitution model. (c) Effective population size of global ST764 estimated from the time-calibrated tree shown in panel (b).

Ancestral state reconstruction suggests that the bacteria emerged in Japan and underwent local diversification there from the early 1980s through the 2000s before disseminating to other countries (Fig. 3b). The analysis inferred three transmission events from Japan to China during the late 1990s and early 2000s and one transmission event to Thailand in the early 2000s (Fig. 3b). Following transmissions to China or Thailand, the bacteria appear to have undergone local expansion within each country. Notably, within the Thailand-associated clade (tMRCA 2001; 95% CI: 1993, 2010), a subclade comprising samples from the UK, Russia, the Netherlands and Australia was identified, with a tMRCA also estimated in the early 2000s (tMRCA 2002, 95% CI: 1997, 2011), indicating onward dissemination from this lineage to multiple non-Asian countries. In addition, a monophyletic clade of Japanese ACME-positive samples was identified, with a tMRCA estimated in the early 1990s. This subclade was inferred to have circulated locally in Japan, with no further cross-country transmission observed. The absence of ACME-positive ST764 isolates reported after 2020 suggests that this lineage may not have undergone further expansion.

Furthermore, the dynamics of the bacterial effective population size were estimated from the time-calibrated phylogeny using the maximum-likelihood skygrid model, which inferred a rapid increase from the early 1980s through the late 1990s, followed by a plateau between the early 2000s and mid-2010s and a sharp decline thereafter (Fig. 3c).

## Discussion

In this study, we first report a complete genome of SATU136, an MRSA ST764 SCC*mec* type II isolate from Thailand. The isolate was from the blood of a subsequently deceased 80-year-old patient in 2014. MRSA ST764 SCC*mec* type II originally described in Japan contained an ACME element. However, upon investigation of SATU136, only the normal chromosomal *arc* gene cluster was identified distant from SCC*mec*, and the ACME-associated *arc* gene cluster was absent.

The ACME previously reported in MRSA ST764 retained the *arc* gene cluster almost identical to USA300 but lacked the *opp* clusters and raised the concern that the genotype may be transmitted in the community [13] and become widespread finally [3,47]. MRSA ST764 was indeed reported more commonly in Japan [8,48] over time. It is, therefore, surprising to find that only a small fraction of MRSA ST764 in Japan, belonging to the single clade, harboured ACME. This suggests that most MRSA ST764 strains in Japan as well as other countries are probably transmitted in hospital settings.

The evolutionary analysis confirms the hypothesis of the origin of MRSA ST764 in Japan, where the strains have become common. Up to present, considerable numbers of MRSA ST764 cases were reported additionally only in China and Thailand. The topology of the tree indicated that the transmissions to China and Thailand were limited to only a few times, probably one-way, and by ACME-negative strains. Once reaching China and Thailand, there has been no evidence that the strains have been transmitted back to Japan, although there might be transmission to other countries. Although the lack of ACME conformed to the fact that most reported ST764 infections in Thailand and China were in hospitalized patients, this could be a sample bias and community transmission might still be possible, presumably facilitated by other mechanisms. A recent study of ear infections in China suggested that community transmission of MRSA ST764 did occur in China, possibly attributable to the extensive quinolone-resistant phenotype [34].

MRSA ST764 was identified only occasionally in Australia, the Netherlands, Russia and the UK. In almost all cases, the etiologic strains were embedded in the clade from Thailand, suggesting the transmission from Thailand. The genomes of both UK isolates were highly similar, suggesting a possible transmission in the UK. The last isolate from the Netherlands was embedded in a Chinese clade. Whether ST764 would establish continuous transmission in the countries remains to be investigated.

MRSA ST764 was probably carried across countries by asymptomatic carriers. The rates of MRSA carriage were usually reported to be lower than those of methicillin-sensitive *S. aureus* [49]. The carrier rates of HA-MRSA in the community were usually less than the rates in hospitals but can still be sometimes considerable [50]. The roles of asymptomatic carriers in propagating HA-MRSA outside or across hospitals deserve more investigations. Better knowledge may lead to a control measure that restricts MRSA strains geographically.

Although some MRSA ST764 may carry SCC*mec* type IV, most carry SCC*mec* type II, similar to ST5, from which it is derived. However, structural variants of the SCC*mec* type II exist, probably due to the recombination of two IS*257* elements presented in ST5, resulting in the deletion of the 5-kb-long segment in ST764. This deletion was also revealed in MRSA ST764 isolates from China [7]. IS257 has been involved in the deletion of genome fragments in SCC*mec* as well as other genomic regions of *S. aureus* [51].

Although there have been considerable concerns about the threat of MRSA ST764, the calculated effective population size did not suggest any further expansion at present. The expansion of the genotype was reported only in Japan, China and Thailand. The isolates in China and Thailand were apparently derived from Japan. After the initial cross-country transmission, the expansion of the transmitted clade was mostly restricted to the country. This suggests that a genomic surveillance programme of MRSA may be useful for early detection and facilitate early rigorous control of ST764, particularly in hospital settings in countries where the strain is still not existing.

This study, nevertheless, was limited by the availability of data. Many countries may not have a system for genetic surveillance of MRSA, and the presence of MRSA ST764 is likely to be underreported. This, together with its reported high virulence and possibly expansion as demonstrated in Japan, warrants proper surveillance of MRSA ST764.

## Supplementary material

10.1099/mgen.0.001774 Supplementary Material 1.

10.1099/mgen.0.001774 Supplementary Material 2.

## Abbreviations

ACME: arginine catabolic mobile element
CA-MRSA: community-acquired methicillin-resistant *Staphylococcus aureus*
CARD: Comprehensive Antibiotic Resistance Database
CDS: coding sequence
DR: direct repeat
HA-MRSA: hospital-acquired methicillin-resistant *Staphylococcus aureus*
IS: insertion sequence
MPPA: marginal posterior probability approximation
MRSA: methicillin-resistant *Staphylococcus aureus*
PVL: Panton–Valentine leukocidin
RGI: Resistance Gene Identifier
SCCmec: staphylococcal cassette chromosome mec
ST: sequence type
UFBoot: ultrafast bootstrap
VFDB: Virulence Factor Database
