# Supplementary material for: Phylogenomic analysis of a methicillin-resistant Staphylococcus aureus ST764 isolate from Thailand in global context reveals limited cross-border transmission
Source: Microb Genom. 2026 Jul 16;12(7):001774. doi: 10.1099/mgen.0.001774 (PMC13374725; doi:10.1099/mgen.0.001774)
Supplement: Supplementary Material 1. [file mgen-12-01774-s001.pdf]

Supplementary Figures

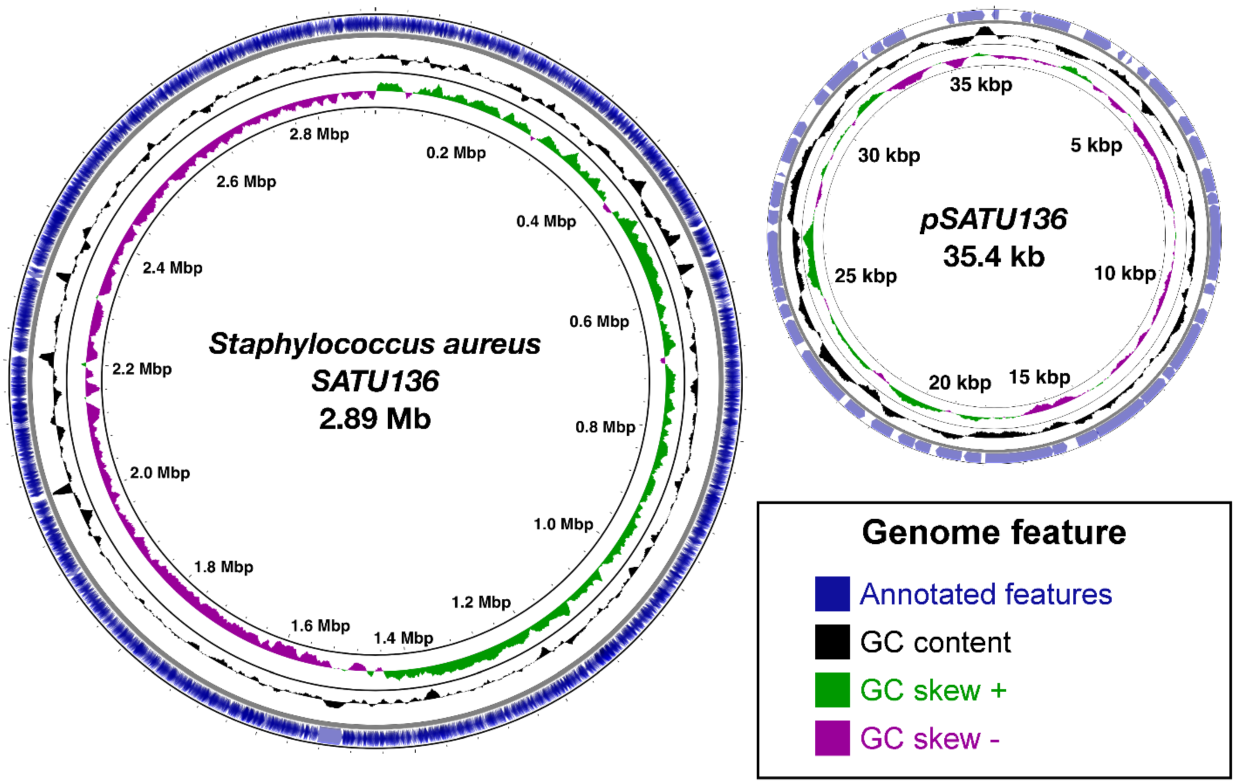

Figure 1: Genomic map of the *S. aureus* SATU136 chromosome and plasmid. The first and second outmost circles show coding sequences (CDSs) on the plus and minus strands. The third and fourth circles show GC content and GC-skew, respectively. Colours are explained in the inset legend.

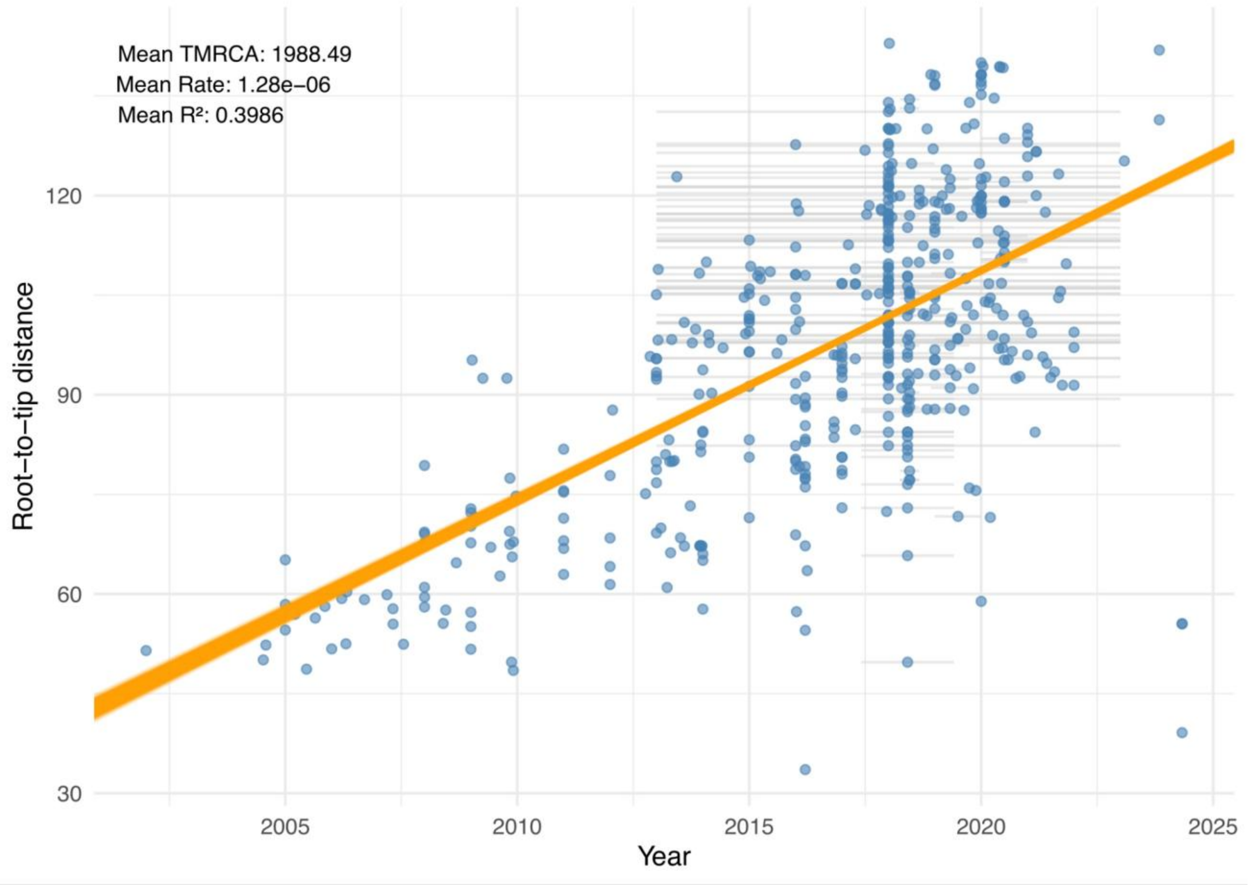

Supplementary Figure S2. Root-to-tip regression. For samples with a date range, a single point randomly drawn from within the range was used as the tip date at each iteration. For three samples with unknown collection dates, the public release date of the genome data was used as a fixed-point estimate for this temporal signal analysis (proper tip dates for these samples were subsequently estimated with LSD2). Randomization was performed over 1,000 iterations. The 1,000 resulting regression lines are shown in orange. Each steelblue point shows a sample plotted at the midpoint of its date range against its root-to-tip genetic distance; horizontal grey bars indicate the date range for samples with date uncertainty.

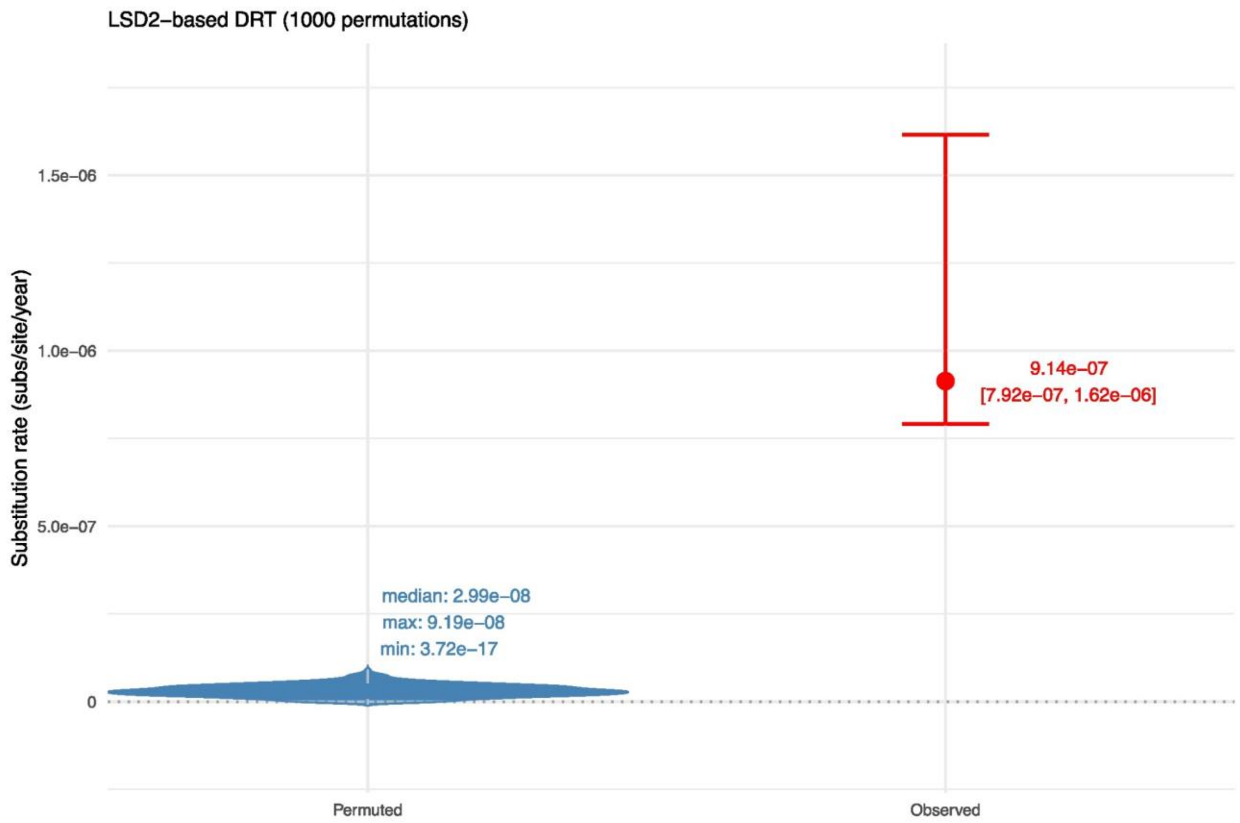

Supplementary Figure S3: Date-randomization test (DRT) for temporal signal. Distribution of substitution rates from 1,000 LSD2 runs with permuted tip dates (blue violin; blue point = median) compared to the rate estimated from the observed dates (red point with 95% confidence interval). The observed rate is approximately 10-fold higher than the maximum permuted rate, and its 95% CI does not overlap with the permuted distribution, confirming a robust temporal signal.

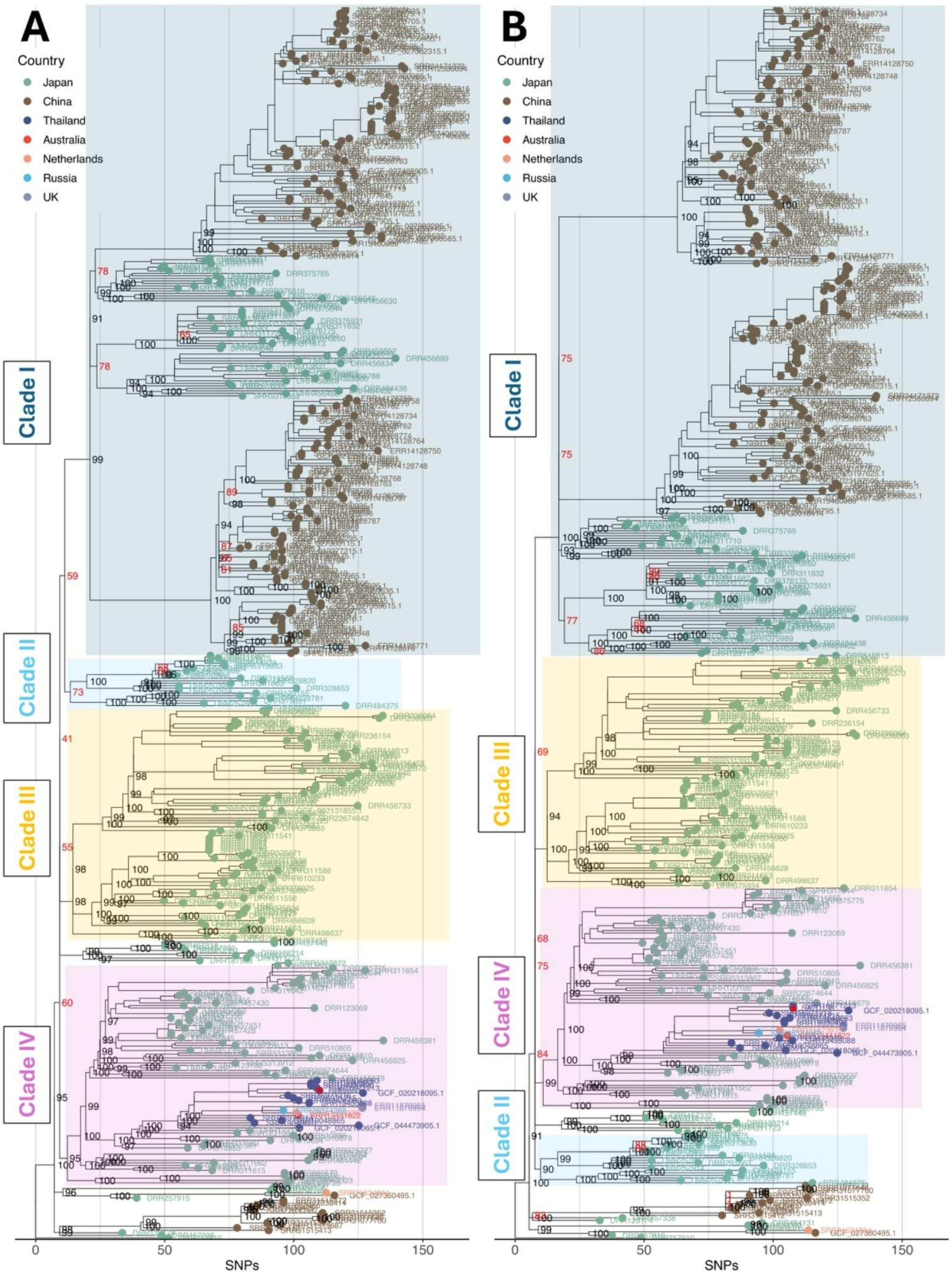
A
B
Country
Japan
China
Thailand
Australia
Netherlands
Russia
UK
Clade I
Clade II
Clade III
Clade IV
SNPs
0
50
100
150

Supplementary Figure S4: Maximum-likelihood phylogenies of 493 *S. aureus* ST764 isolates, inferred from recombination-masked SNP alignments generated using SATU136 (**A**; whole-genome alignment length: 2,898,311 bp; SNP alignment length: 10,769) and KUH180062 (**B**; whole-genome alignment length: 2,939,465 bp; SNP alignment length: 10,776) as the references. The tree was rooted using MRSA ST5 (Mu50) as an outgroup, which was subsequently dropped. Phylogenetic clades were assigned based on the basal branching pattern and UFBoot scores. Internal node labels show UFBoot support values from 1,000 ultrafast bootstrap pseudoreplicates, displayed only at deep internal nodes to reduce visual clutter. Values < 90 are shown in red. Tip points show the country of origin. Red star, SATU136.

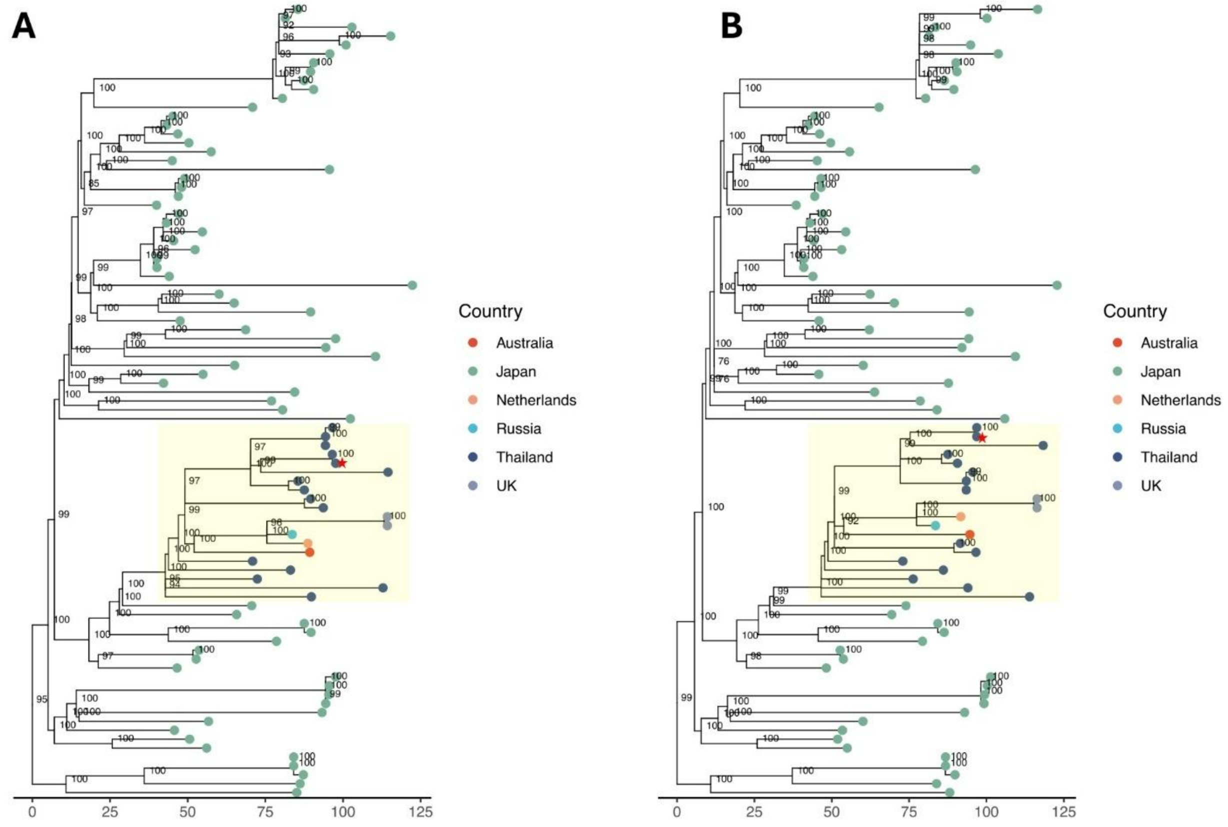

Supplementary Figure S5. Detailed view of clade IV from the maximum-likelihood phylogenies in **Supplementary Figure S4**, inferred from whole-genome alignments using SATU136 (**A**) and KUH180062 (**B**) as reference genomes. Tip points are colored by country of collection, and internal node labels show ultrafast bootstrap (UFBoot) support values based on 1,000 pseudo-replicates. Red star, SATU136.

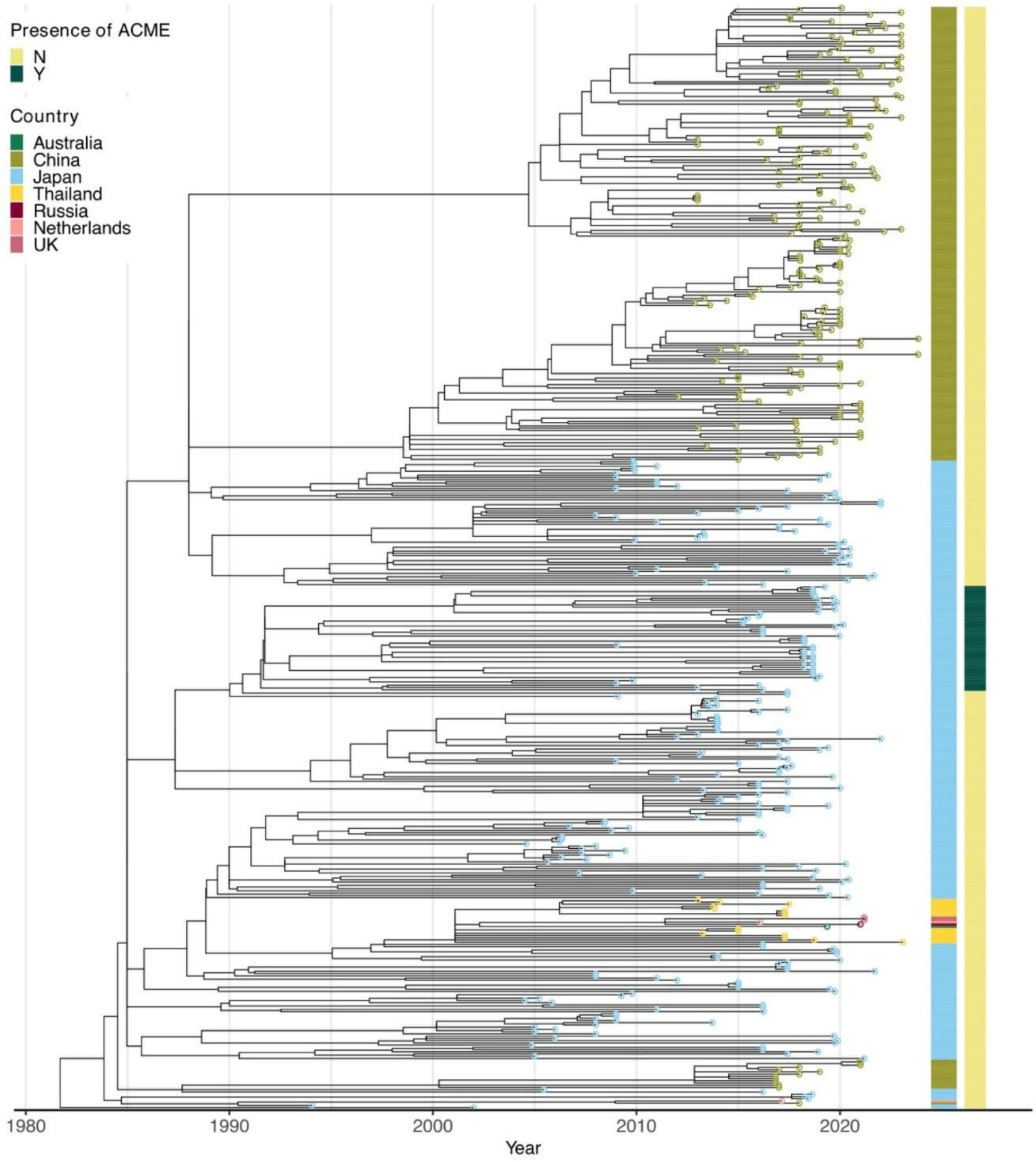

Supplementary Figure S6. Time-calibrated phylogeny inferred using KUH180062 as the reference genome. Whole-genome alignment was generated with Snippy using KUH180062 (Japan) as the reference, followed by maximum-likelihood tree reconstruction with Gubbins (with recombination removed). The resulting tree was time-calibrated with LSD2 following the same approach used for the main analysis (**Figure 3**, which used SATU136 as the reference).
